# Deletion of a putative HDA-1 binding site in the *hlh-2* promoter eliminates expression in *C. elegans* dorsal uterine cells

**DOI:** 10.17912/micropub.biology.000449

**Published:** 2021-09-02

**Authors:** Taylor N Medwig-Kinney, Nicholas J Palmisano, David Q Matus

**Affiliations:** 1 Stony Brook University

## Abstract

The helix-loop-helix transcription factor *hlh-2* (E/Daughterless) has been shown to play an important role in regulating cell fate patterning, cell cycle, and basement membrane invasion in the context of the development of the *C. elegans* somatic gonad. Here, using CRISPR/Cas9 genome engineering, we generated a new *hlh-2* allele (*hlh-2(Δ-1303-702)*) in the endogenous, GFP-tagged *hlh-2* locus. This allele represents a deletion of a 601 bp region in the *hlh-2* promoter that contains a putative binding site of the histone deacetylase *hda-1* (HDAC) according to publicly available ChIP-sequencing data. Strikingly, we find that HLH-2 expression is virtually absent in the dorsal uterine cells of *hlh-2(Δ-1303-702)* animals compared to wild type controls. Levels of HLH-2 in the anchor cell and ventral uterine cells are only modestly reduced in the mutant; however, this does not seem to be functionally significant based on the lack of relevant phenotypes and expression levels of a downstream gene, NHR-67 (TLX/Tailless/NR2E1), in these cells. Taken together, these results support growing evidence that HDACs can potentially positively regulate transcription and provide a new reagent for studying *hlh-2* regulation.

**Figure 1. Characterization of new allele deleting putative HDA-1 binding site from the hlh-2 promoter:**
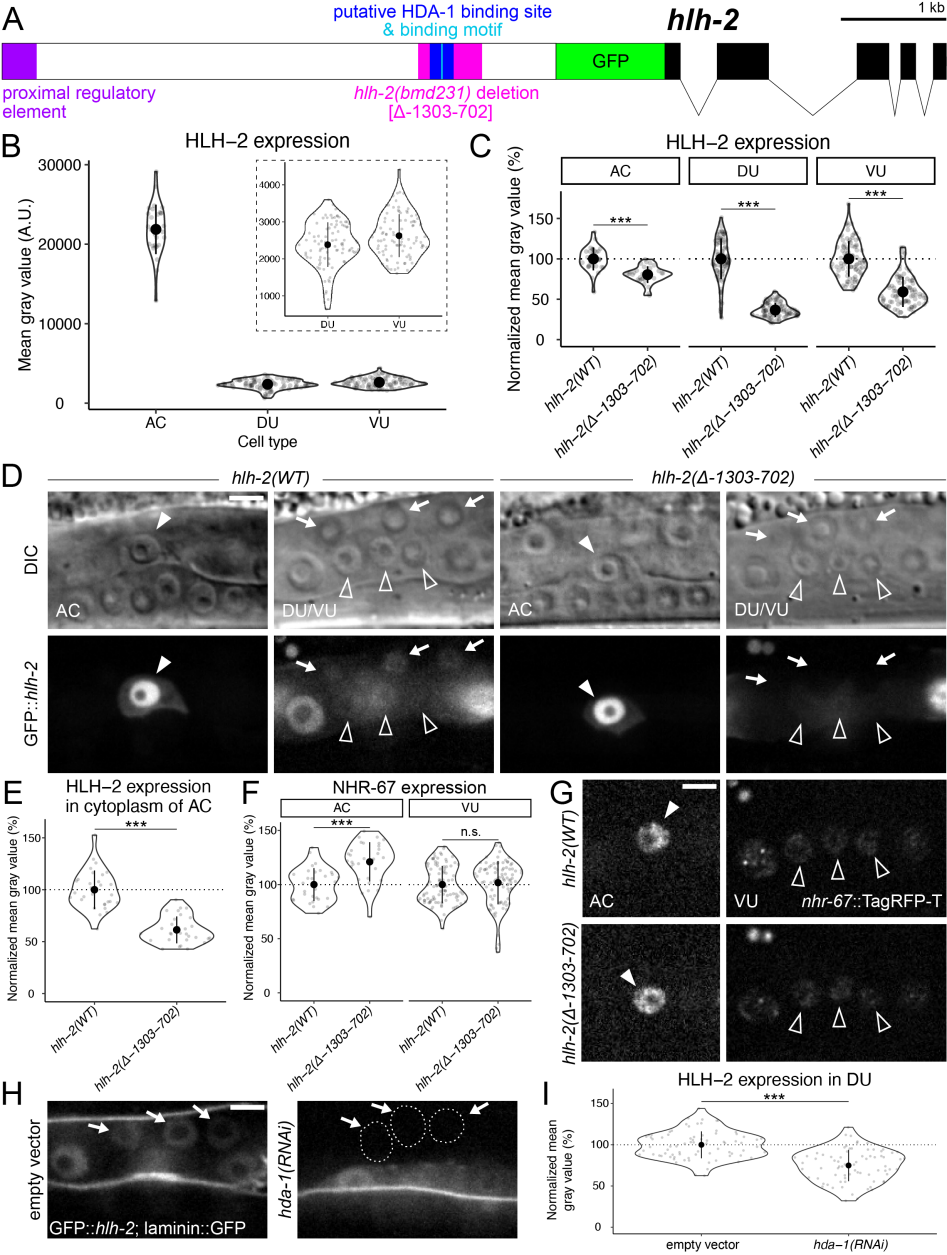
(**A**) Schematic of the endogenous GFP::*hlh-2* locus and promoter region, annotated with a previously characterized proximal regulatory element, putative HDA-1 binding site (based on published ChIP-sequencing data) and binding motif, and the genomic region deleted in the *hlh-2(bmd231)* mutant. (**B**) Nuclear expression of wild type (wt) GFP::*hlh-2* in the anchor cell (AC), dorsal uterine (DU) cells, and ventral uterine (VU) cells. (**C-D**) Nuclear expression of GFP::*hlh-2* in the AC (solid arrowhead), DU (arrows), and VU (unfilled arrowheads) cells of *hlh-2(WT)* and *hlh-2(Δ-1303-702)* animals. (**D-E**) Cytoplasmic expression of HLH-2 in the AC of *hlh-2(WT)* and *hlh-2(Δ-1303-702)* animals. (**F-G**) Expression of *nhr-67*::TagRFP-T in *hlh-2(WT)* and *hlh-2(Δ-1303-702)* animals. (**H-I**) Levels of GFP::*hlh-2* following depletion of *hda-1* via RNA interference compared to empty vector control in the DU (nuclei outlined *hda-1(RNAi)* micrograph). Statistical significance determined through using an unpaired two sample t-test, with Welch’s correction (*p ≤ 0.05; ***p ≤ 0.001; n.s., not significant). Scale bars: 5 µm.

## Description

During *C. elegans* development, two cells, Z1 and Z4, give rise to the entire somatic gonad, including populations of dorsal uterine (DU) and ventral uterine (VU) cells, as well as the postmitotic anchor cell (AC), which invades the underlying basement membrane during uterine-vulval morphogenesis (Kimble & Hirsh, 1979; Sherwood & Sternberg, 2003). The helix-loop-helix transcription factor *hlh-2* (E/Daughterless) is expressed in these three cell types, among others, and is particularly enriched in the AC following post-transcriptional down-regulation of HLH-2 in the VU through dimerization-driven degradation (Karp & Greenwald, 2003; Sallee & Greenwald, 2015). At the L2 stage, HLH-2 regulates the Notch signaling event that specifies AC and VU fates, with its initial onset predicting the lineage ultimately giving rise to the presumptive VU (Karp & Greenwald, 2004; Attner *et al.*, 2019). Following AC/VU specification, HLH-2 functions to regulate cell cycle arrest and invasion of the AC (Medwig-Kinney *et al.*, 2020; Schindler & Sherwood, 2011). We and others have shown that HLH-2 does so by regulating expression of the nuclear hormone receptor and transcription factor NHR-67 (Tailless/TLX/NR2E1), and likely cell-cycle independent, pro-invasive targets as well (Bodofsky *et al.*, 2018; Medwig-Kinney *et al.*, 2020).

In addition to *hlh-2* and *nhr-67*, the histone deacetylase (HDAC) *hda-1* was also identified as a regulator of AC invasion through a reverse genetic screen (Matus *et al.*, 2010). It was later shown that *hda-1* function is necessary for AC invasive fate differentiation and plays a role in the pro-invasive pathway encompassing NHR-67 (Matus *et al.*, 2015). In order to further elucidate the mechanism by which HDA-1 regulates AC invasion, we sought to test we sought to test whether HDA-1 regulated expression of *hlh-2*. Previous studies of the *hlh-2* promoter identified a proximal regulatory element (*hlh-2(prox)*) that confers expression in the AC and VU cells specifically (**Figure 1A**) (Sallee & Greenwald, 2015). Approximately 3.3 kb downstream of this *hlh-2(prox)* element is an HDA-1 binding motif that lies within a putative HDA-1 binding site based on ChIP-sequencing data generated by the modENCODE Project (**Figure 1A**) (Shao *et al.*, 2020; Celniker *et al.*, 2009). Using CRISPR/Cas9 genome engineering, we edited the endogenous *hlh-2* locus to introduce a deletion mutation (*hlh-2(bmd231)*) from 1303 to 702 base pairs (bp) upstream of the GFP start codon, hereafter referred to as *hlh-2(Δ-1303-702)* (**Figure 1A**).

Consistent with findings using immunostaining, we find that endogenous GFP::*hlh-2* expression is normally enriched in the AC compared to the VU and DU (Karp & Greenwald, 2003) (**Figure 1B**). When compared to wild type, the *hlh-2(Δ-1303-702)* mutant showed a modest but statistically significant reduction in HLH-2 expression in the AC and VU cells (**Figure 1C-D)**. The endogenous GFP::*hlh-2* allele paired with high resolution microscopy allowed us to detect subtle regulation of HLH-2 expression by *hda-1* in the AC and VU cells that was not previously reported using a transgenic reporter (Ranawade *et al.*, 2013). DU expression, however, was virtually eliminated in the mutant when accounting for camera-derived background noise (**Figure 1C-D)**. We also observed that cytoplasmic expression of HLH-2 in the AC was modestly reduced in the mutant as well (**Figure D-E**). However, the alterations in HLH-2 expression in the AC and VU do not seem to be functionally significant, as expression of a NHR-67, a downstream target of HLH-2 in the cell cycle dependent pro-invasive gene regulatory network (Medwig-Kinney *et al.,* 2020), was not significantly reduced (**Figure 1F-G**). Furthermore, no defects in AC specification or invasion were observed in any of the strains containing the *hlh-2(Δ-1303-702)* allele (n > 50) based on both the presence of a single AC and an underlying gap in the basement membrane, in all animals examined (visualized by *cdh-3p*::mCherry::moeABD and laminin::GFP, respectively). RNAi-induced knockdown of *hda-1* resulted in reduced levels of HLH-2 in the DU, providing further evidence of this regulatory interaction (**Figure 1H-I**).

In summary, here we generate and characterize a new mutant allele of *hlh-2*. Deletion of the genomic region containing a putative HDA-1 binding site from the *hlh-2* promoter appears to primarily affect expression in the DU cells and is recapitulated through RNAi-induced knockdown of *hda-1*. Although HDA-1 is traditionally thought of as a repressor of transcription through chromatin deacetylation (Kadosh & Struhl, 1997; Kadosh & Struhl, 1998; Rundlett *et al.*, 1998; Gui *et al.*, 2003), this data adds to the growing evidence that HDACs can also function as activators of transcription (Vidal & Gaber, 1991; De Nadal *et al.*, 2004; Wang *et al.*, 2014). Whether there is a functional consequence of *hlh-2* depletion in the DU cells is currently unknown. It is our hope that this reagent will be useful to the *C. elegans* community to further study the roles of *hda-1* and *hlh-2*.

## Methods

**Strain maintenance:**

Animals were reared under standard conditions and cultured at 25°C (Brenner, 1974). Animals were synchronized through alkaline hypochlorite treatment of gravid adults to isolate eggs (Porta-de-la-Riva *et al.*, 2012). The RNAi clone targeting *hda-1* was generated by cloning 923 bp of cDNA (available from wormbase.org; Harris *et al.*, 2020) into the highly efficient T444T RNAi feeding vector (Sturm *et al.*, 2018). RNAi experiments were performed by feeding synchronized L1s following hypochlorite treatment.

**CRISPR/Cas9 injections:**

In order to generate the deletion mutation in the *hlh-2(Δ-1303-702)* allele, Cas9 protein injections were performed as previously described (Paix *et al.*, 2014; Ghanta *et al.*, 2020) with minor modifications to the published protocols. In short, a 200 bp ssODN donor repair ultramer for *hlh-2* (IDT) was constructed. To facilitate easy screening of our edit, a PvuI restriction site was engineered into the ssODN repair oligo as previously described (Paix *et al.*, 2014), flanked on each side by 94 bp of sequence homologous to the region surrounding the putative HDA-1binding site. Single guide RNAs for *hlh-2* and *dpy-10* (co-CRISPR marker) were purchased from CRISPRevolution by Synthego (Synthego Corporation). The Cas9/sgRNA ribonucleoprotein complex was formed by incubating 30 pmols of Cas9 NLS protein (California Institute for Quantitative Biosciences at UC Berkeley (QB3-Berkeley)) with 23.75 pmoles of each *hlh-2* gRNA, and 47.5 pmoles of *dpy-10* sgRNA for 15 minutes at 37°C. The *hlh-2* and *dpy-10* ssODN donor repair ultramers (IDT) were then added to the reaction at a final concentration of 2.2 µg.

TagRFP-T::AID was inserted into the C-terminus of the endogenous *nhr-67* locus via CRISPR/Cas9 mediated genome engineering using the self-excising cassette method (Dickinson *et al.*, 2013; Dickinson *et al.*, 2015). The sgRNA targeting sequence in pDD122 was replaced with the sgRNA targeting the C-terminus of *nhr-67* using Gibson cloning (Dickinson *et al.*, 2013). The repair template for *nhr-67* was generated by cloning homology arms, synthesized by Twist Biosciences (left homology arm) and by PCR using genomic DNA as a template (right homology arm), into pTNM063 (TagRFP-T::AID repair template) (Ashley *et al.*, 2021).

**Live cell imaging**:

Micrographs were collected on a Hamamatsu Orca EM-CCD camera mounted on an upright Zeiss AxioImager A2 with a Borealis-modified CSU10 Yokagawa spinning disk scan head (Nobska Imaging) using 488 nm and 561 nm Vortran lasers in a VersaLase merge and a Plan-Apochromat 100×/1.4 (NA) Oil DIC objective. MetaMorph software (Molecular Devices) was used for microscopy automation. Animals were mounted into a drop of M9 on a 5% Noble agar pad containing approximately 10 mM sodium azide anesthetic and topped with a coverslip.

**Image quantification:**

Images were processed using Fiji/ImageJ (v.2.0.0) (Schindelin *et al.*, 2012). Nuclear expression of HLH-2 and NHR-67 was quantified by measuring mean gray value as previously described (Medwig-Kinney *et al.*, 2020). DU and VU cells were sampled by selecting the three most proximal to the AC and closest to the coverslip. Cytoplasmic expression of HLH-2 was measured using the freehand selection tool, tracing the outline of the cell but excluding the nucleus. For all quantification, background subtraction was performed by subtracting the mean gray value of a background region of an equal area to account for EM-CCD camera noise.

**Data visualization and statistical analysis:**

Representative micrographs were processed using Fiji/ImageJ and assembled into figures using Adobe Illustrator (v.23.0.6). RStudio (v.1.4.1717) was used to generate violin/sina plots and to perform statistical analyses.

## Reagents

**Strains:**

**Table:** 

**Strain**	**Genotype**	**Source**
DQM350	*hlh-2*(bmd90[*hlh-2p*::LoxP::GFP::HLH-2]) I; *qyIs225*[*cdh-3p*::mCherry::moeABD] V; *qyIs7*[laminin::GFP] X.	Medwig-Kinney *et al.,* (2020)
DQM704	*nhr-67(bmd212*[*nhr-67p*::NHR-67::TagRFP-T::AID]) IV; *hlh-2(bmd90*[*hlh-2p*::LoxP::GFP::HLH-2]) I.	This study
DQM785	*hlh-2(bmd231*[*hlh-2p(Δ-1303-702)*>LoxP::GFP::HLH-2]) I; *qyIs225*[*cdh-3p*::mCherry::moeABD] V; *qyIs7*[laminin::GFP] X.	This study
DQM900	*hlh-2(bmd231*[*hlh-2p(Δ-1303-702)*::LoxP::GFP::HLH-2]) I; *nhr-67(bmd212*[*nhr-67p*::NHR-67::TagRFP-T::AID]) IV.	This study

**Sequences:**

**Table:** 

**Reagent**	**Sequence**
*dpy-10* sgRNA	GCUACCAUAGGCACCACGAG + Synthego modified EZ Scaffold
*dpy-10* ssODN	CACTTGAACTTCAATACGGCAAGATGAGAATGACTGGAAACCGTACCGCATGCGGTGCCT ATGGTAGCGGAGCTTCACATGGCTTCAGACCAACAGCCTAT
*hda-1(RNAi)* targeting sequence	TTGATGGAGCACGGTAAGCGCCGTGTCGCCTACTACTATGACTCCAACATTGGAAATTACTA TTATGGTCAAGGGCACGTCATGAAGCCACATCGTATCAGAATGACCCATCATCTCGTTCTCA ACTATGGTCTGTACCGGAATTTAGAGATTTTCCGCCCATTCCCTGCATCATTCGAAGACATG ACTCGTTTTCACAGCGACGAGTACATGACGTTTTTGAAGAGTGCGAATCCAGATAATCTGA AATCCTTCAACAAACAAATGCTTAAGTTCAATGTTGGAGAAGATTGTCCTCTCTTTGATGG TCTTTATGAGTTCTGCCAACTCAGTTCGGGAGGTTCTCTGGCTGCTGCCACTAAATTGAAC AAGCAGAAGGTGGACATTGCTATCAATTGGATGGGAGGCCTCCATCACGCCAAGAAAAG CGAGGCGTCCGGATTCTGTTACACCAATGACATCGTTCTCGGTATTCTCGAGCTTCTCAAG TACCACAAGCGAGTACTTTACGTCGATATTGATGTTCATCACGGAGATGGAGTAGAGGAG GCGTTCTATACGACTGATCGAGTAATGACAGTGTCATTCCATAAATATGGAGATTTCTTCCC AGGAACCGGAGACCTGAAAGATATAGGAGCTGGAAAAGGAAAGCTCTATTCAGTCAATG TTCCACTTCGCGATGGAATCACCGACGTCTCTTACCAGAGTATTTTTAAACCAATCATGAC AAAGGTTATGGAGAGATTTGATCCCTGTGCTGTTGTTCTTCAATGTGGAGCTGATTCTCTC AACGGAGATAGACTTGGACCATTCAATCTGACCTTGAAAGGCCACGGAGAATGTGCTCG TTTCTTCCGAAGCTACAACGTTCCACTTATGATGGTCGGTGGAGGTGGATACACTCCAAG AAATGTGGCACG
*hlh-2* (-1303 bp) gRNA	AUGAAUGUACUCCCUACAGU + Synthego modified EZ Scaffold
*hlh-2* (-702 bp) gRNA	UAAGGAUUCGUAAACAUUGU+ Synthego modified EZ Scaffold
*hlh-2(Δ-1303-702)* ssODN	CTCTCACTCTTACCATATTCTGAAGAATTAAAATTTCAGAGATCCCTACAAAACTCTAATAAC ATGCTTCAAAAATGAATGTACTCCCTACAGTCGATCGTAATAGACAATGTTTACGAATCCTT ACCAATTTTGAATTTAAACAAGAACGCAAATGTATTGTAGGGCAGTTTTTTTTTCAATTATT GAGTTTATCAAAA
*nhr-67* C-terminus sgRNA	AGAGAGTGTTAATGTTGAAG
*nhr-67* C-terminus left homology arm	GGAATAATGTGAGACTTCACTATAAAGGTAAACGCTGTTTTTCTGAGTGGGTTGCAACGATC AAAGTTAATTAAATATTGTATTTGCTAGTTTGAAGGTTGCTAATTCTTTTTTAAAATTAATTAA TTAACCAATTGAAAAAGTTCATTTATAGTTTTTGTACGATTATCCTATTCAAAAGTTCATTTTT CGGCTCAAAATATAAAAAATTCCACAATTAAAAAAGCAGTGTTTTTTGTTCTCACAAAAAAT GCAAATTATTTCCTATTTGCTTAAAAAGCGAAATTGATTATTGAAAACTAATGAAAACTAAA CGCTCTAGTACCATCTTTCCTTCTGAAAACTTCACAGTCTGAAACCTATTTCAGATGTATAAA TGCAATTGCCGCTATTCCAACAACTTCAATAATTGATGTTCTATTCCGCCCTTCAATTGGATC AGCTTCAATGCCAAGACTTATTCAAGACATGTTCAAGCCACCACAACAACCCACTCCTACG TCACTGTTTCCAATGGCAAACTTCAATTTGAACTTTCTATTAAAACAAGAAAAAACCGAAA CTGAAGAGGGTGAAGATATTGAAGAAGAGGATGATGCGACGAGTAGCAATCAATTTGATG AAAATTCTTCTACTGATGATAGGTATGATGAGCATTTATTAGTACAGTGGATTAACTGAGTC TGTTGCAGATCTGTCGGAGAACTGGATCCCGTTCAACTTTTCTTGGCTCTTAATTCCTCAAC TCAGCCTTCATCGGCATCATCCCCTTCCTCTTCAAGACCACGTCATTCGATTCGATCAATAAC TGAATTATTATCAATTCAAGAAGAGGAAAGCGTGAACGTGGAGGAAGTG
*nhr-67* C-terminus right homology arm	TAAATAGTAAATTCATGTTTCATATACAGTAACTCAATTATTCTAAGTATCTCTTTTCATTGTCT TTTTCACTCCGTTTCTTGCCTCGCCCGGATTTTCATTGGATTTTGATTTATACTTTCAAAATTT CATTTTTCAATTGTAAATTTTTAATTTAAATTTAGAGAAAAAAAATGGTAAGCTTTAAAACTA ATTTATTATTTTCTGATTTATATTGTGAACAGATGAATAAAACGTTTACAACAATGGCAATTG GCATACAAACATCATTAAAAAAAGGTGAATCATACAGTTTTGAGAAGACTACATATGATTCG AAACATAAAAAACAATAGAAATCAATAAATGATGGGAGAGAAACCGAGAGATTTATTGGAA AATGGAACGGTTGAATGTCATATTGTGTCATCGTCGTTTTCCTTGTTAATGGCCTCGTTTCTT ATCATATCCATATCATTCTGATATTCCCATTCAACTAGATGACCAAATCATTAGCTGACTGATT TCCCCCTTTTTAATGATTTGTTTATGGTTTTGTCAATAGTTTGAACGCGTTACGTTTTTGTAGA TAAACCTGATTATGCTAGAATATTTTTTATTGAGATTTATTGACTAATTTAAAGTTTTAAACTA ACAGAACAACAACCAATATTATAAAGAAAATGCTTTAAACTCATTTTTGAACATCCAGAACA TCCGAAAAATAACCAACATGGTTAAGATTTTTCAAAGTTTTTAGTCAAAGTTTCGGTATCTAT TTGCAATTTTCAAAAAACATGGAATATTTTCAGTAATTGCTTTTTCGAACTCCCAGACTGTTT GAATACAAAAATTGAAAAGCAAGTAAACAATAAAAAATTGTAGATATTTTTTCAAAGACTTT CAAAATTATAGGCGTAGGCTTCACTAATTTTTGACTGTCAGTAAAATATTTATTTCAAAAAAA TTTTAAAAGTTTTACCATAATATTTGGGCATGGGCATTTTACTTTTAAAAACGATTTCTAAAG AAACCATTTTTATATGTAAAACAGTTTTGCTCAATTTTACCAGTTATCAA
Forward primer to amplify *nhr-67* C-terminus right homology arm from gDNA	CGGCGGCGTTCGTGAAATAAATAGTAAATTCATGTTTCATATAC
Reverse primer to amplify *nhr-67* C-terminus right homology arm from gDNA	GCTATGACCATGTTATCGATTTCCTAGTTGATAACTGGTAAAATTGAGCA
